# Multivariate analysis and digital twin modelling: Alternative approaches to evaluate molecular relaxation in photoacoustic spectroscopy

**DOI:** 10.1016/j.pacs.2023.100564

**Published:** 2023-10-09

**Authors:** A. Zifarelli, A.F.P. Cantatore, A. Sampaolo, M. Mueller, T. Rueck, C. Hoelzl, H. Rossmadl, P. Patimisco, V. Spagnolo

**Affiliations:** a PolySense Lab, Dipartimento Interateneo di Fisica, University and Politecnico of Bari, Via Amendola 173, 70126 Bari, Italy; b PolySense Innovations S.R.L. via Amendola 173, Bari, Italy; c Sensorik-ApplikationsZentrum (SappZ), Regensburg University of Applied Sciences, 93053 Regensburg, Germany; d Institute of Analytical Chemistry, Chemo, and Biosensors, University of Regensburg, 93053 Regensburg, Germany; e Thorlabs GmbH, Münchner Weg 1, 85232 Bergkirchen, Germany

**Keywords:** relaxation effects photoacoustic spectroscopy, multivariate analysis, digital twin model, QEPAS sensor, methane detection over a wide concentration range, water vapor influence on relaxation, Partial Least Squares Regression

## Abstract

A comparative analysis of two different approaches developed to deal with molecular relaxation in photoacoustic spectroscopy is here reported. The first method employs a statistical analysis based on partial least squares regression, while the second method relies on the development of a digital twin of the photoacoustic sensor based on the theoretical modelling of the occurring relaxations. Methane detection within a gas matrix of synthetic air with variable humidity level is selected as case study. An interband cascade laser emitting at 3.345 µm is used to target methane absorption features. Two methane concentration ranges are explored targeting different absorptions, one in the order of part-per-million and one in the order of percent, while water vapor absolute concentration was varied from 0.3 % up to 2 %. The results achieved employing the detection techniques demonstrated the possibility to efficiently retrieve the target gas concentrations with accuracy > 95 % even in the case of strong influence of relaxation effects.

## 1 Introduction

Gas sensing technologies based on optical spectroscopy have been widely investigated in the past decades with the aim of developing reliable sensors to be operated in real-world applications [1], [2]. In the large panorama of available detection techniques, photoacoustic spectroscopy (PAS) represents a well-consolidated technology, characterized by versatility, robustness, and sensitivity. PAS demonstrated the detection of different gas species for several applications, as environmental monitoring [3], [4], human healthcare [5], [6], and industrial processes control [7], [8]. In PAS, modulated optical radiation is absorbed by the target analytes and converted into acoustic waves are generated by photoacoustic effect. Then, the acoustic waves are detected by a transducer which in turn returns an electric signal proportional to the amplitude of the pressure wave [9]. Typically, in standard PAS setup resonant acoustic cells are used to amplify the generated photoacoustic waves and highly sensitive condenser microphones are used for detection. However, in the recent years different detection systems have been investigated, including: optical detection systems, e.g., interferometry or fiber Bragg grating device; MEMS-based devices; and cantilever beams [10], [11], [12], [13]. Quartz-enhanced photoacoustic spectroscopy (QEPAS) was proposed as a development of traditional PAS, exploiting a quartz tuning fork (QTF) as sharply resonant transducer [14]. Compared to the standard PAS setup the QTF acts as both acoustic resonator and electrical transducer, providing smaller footprint and increased ruggedness. Millimetric acoustic resonator tubes are usually coupled with the QTF to amplify the generated photoacoustic waves, acting like organ pipes. In recent years, QEPAS sensors have been widely employed for trace gas sensing, providing high sensitivity and versatility thus making them suitable to target multiple applications [15], [16], [17], [18], [19]. PAS and QEPAS fall within the category of indirect detection techniques, as the gas absorption is evaluated by means of the energy deposited in the sample by the light source and converted into pressure waves. Therefore, the transducer response is independent of the wavelength selected to excite the target analyte, and this characteristic makes this kind of sensors a suitable approach to multi-gas detection relying on broadband laser sources in particular [20]. However, the photoacoustic conversion of incident radiation into acoustic waves depends on gas sample composition both in terms of heat generation and pressure waves propagation [21]. Dealing with trace gas detection, the latter phenomenon has a minor effect on sensors response, while the heat conversion efficiency may be significant. The non-radiative relaxation process leading to heat generation in the gaseous sample is mainly determined by the transfer rate of the vibrational energy of excited target molecules into kinetic or vibrational energy of surrounding molecules, labelled as V-T and V-V relaxation, respectively [22], [23], [24]. Following the absorption of a photon flux with a harmonic modulation at frequency *f*, the influence of energy transfer processes on the photoacoustic waves generation can be expressed by the radiation-to-sound conversion efficiency parameter, labelled *ε* [25]. This parameter ranges from 0 to 1 depending on all the relaxation pathways of the targeted gas mixture, thus is strongly dependent on the energetic levels distribution of the molecules composing the sample [26], [27], [28]. For this reason, intentionally humidifying the gas sample has been widely employed in QEPAS sensing in the past years, since water (H_2_O) is known to prevent incomplete radiation-to-sound conversion due to its promoting effect [29], [30], [31]. More recently, approaches based on multi-gas detection and signal compensation by means of an external detector have been developed to filter out the molecular relaxation dependencies [32], [33]. These methods demonstrated a good efficiency, but the performed calibrations were limited to a narrow range of target gas concentrations as well as a narrow range of humidity levels, sufficiently high to maximize the conversion efficiency *ε* [34]. Aiming to develop a gas sensor for on field measurements capable to operate under different conditions, it is mandatory to employ more sophisticated data analysis techniques to model the sensor’s response.

Recently, two opposite approaches aiming to deal with matrix effects in photoacoustic spectroscopy have emerged, one based on a multivariate analysis (MVA) as Partial Least Squares Regression (PLSR) [35] and one based on a digital twin (DT) of the developed sensor [36]. These methods address the same issue from two completely different perspectives. The PLSR-based approach relies on the statistical evaluation of the cross-correlation induced on QEPAS signal by the matrix effects while the DT-based approach relies on the theoretical computation of all the relaxation processes occurring in the gas sample.

In this work, we report on a compared investigation on the PLSR-based and DT-based data analysis technique with the aim of filtering out the molecular relaxation effects from QEPAS signal. The two approaches are tested on the same experimental datasets, collected targeting gas mixtures composed of methane (CH_4_) and H_2_O in synthetic air. An interband cascade laser (ICL) with central emission wavelength of 3.345 µm (∼2989 cm^−1^) is used as light source to target CH_4_ absorption features. This spectral region is well-known in literature for the strong matrix effects occurring on the photoacoustic signal corresponding to CH_4_ absorptions due to the influence of water vapor and oxygen [23], [26], [33], [34]. Two CH_4_ concentration ranges are explored separately, targeting different absorption features within the laser dynamic range: a “low-concentration range” in the order of part-per-million (ppm), and a “high concentration range” in the order of percent. The two data analysis techniques allowed the retrieval of CH_4_ concentrations within different samples compositions and the comparative analysis provided an outline of the advantages and disadvantages of the presented methods.

## 2 Partial least squares regression

The use of this statistical tool to model spectroscopic systems has already proven to be effective, benefitting from the large number of information acquired by the optical sensors and being able to deal with spectral and non-spectral interference occurring in PAS and QEPAS measurements [35]. Among the different possibilities, MVA represented a solid class of regression and classification algorithms which have been successfully applied to spectroscopic techniques [37]. Partial least squares regression (PLSR) is a MVA technique representing a development of traditional multiple linear regression, developed to deal with noisy and highly correlated data [38], [39]. When applied to optical spectroscopy, this tool has already demonstrated to be a reliable approach to complex gas mixtures [40], [41]. PLSR extends the traditional linear regression model to include correlation effects, mathematically corresponding to collinearity in the matrices. The linear regression model can be expressed in matrix form as ***Y*** = ***XB*** + ***E***, where ***X*** is the matrix containing the experimental acquisitions, i.e., the spectra, ***Y*** the matrix of the physical parameters to be estimated, i.e., the gas concentrations, ***E*** is the residuals matrix, and ***B*** is the matrix of the regression coefficients. To include correlation effects within the matrices, PLSR assumes that the system is described in terms of truly independent factors called latent variables (LVs) or components. LVs are extracted from the matrices ***X*** and ***Y*** by maximizing the covariance matrix ***cov(X,Y)***, thus projecting the matrices into a new vector space described by the LVs. This operation, called *projection on latent structures* allows the algorithm to perform a linear regression on truly orthogonal and independent vectors, thus returning solid regression coefficients and high predictive power [42]. The number of LVs represents an input parameter for the analysis, and thus it must be carefully evaluated to prevent the regression from being affected by under- or over-fitting of the data [43]. The high versatility of the algorithm and the possibility to describe different systems without a priori investigations make this approach a valuable tool for gas sensing spectroscopy.

## 3 Digital twin

The behavior of complex systems with regard to variations of different parameters is often difficult to predict. In 2003, Michael Grieves proposed the concept of a digital twin (DT) to address this challenge, promising many advantages, like better and more realistic predictions resulting in appropriate actions to be taken [44], [45]. The most common application areas of the DT are the manufacturing sector, with keywords predictive maintenance, smart factory and industry 4.0 as well as prognostics and health management (PHM) [46]. Shafto et al. described a DT to be “an integrated multi-physics, multi-scale probabilistic simulation of a […] system that uses the best available physical models, sensor updates […] to mirror the life of its […] twin” [47]. With the intention to improve the reliability of sensor systems, the concept of the DT was applied to a photoacoustic trace gas sensor for the first time in 2023 [36]. The quantities to be considered in view of DT compensation to finally associate an analyte concentration *c*_*CH4*_ with a photoacoustic amplitude *U* are reported in Eq.(1):

(1) $$U∼\left(\gamma -1\right)\;\frac{Q}{f}\;c_{\mathrm{CH}4}\;\sigma \left(\lambda \right)\;P_{0}\;\varepsilon$$

where, *γ* is the heat capacity ratio of the gas mixture, *Q* the quality factor of the resonator, *σ(λ)* the absorption cross section at the selected wavelength, *P*_*0*_ the incident laser power. For this purpose, the quality factor and the resonance frequency of the QTF, as well as the optical power used for photoacoustic signal generation are measured, while the absorption cross-section of the sample at the wavelength of excitation is simulated using HITRAN database. The core of the DT is the algorithm named CoNRad, which allows the calculation of the collision-based non-radiative relaxation efficiency *ε,* as well as the heat capacity ratio *γ* of the gas mixture, considering pressure, temperature, composition of the mixture, and the laser modulation frequency [48]. According to the definition used by Shafto et al., CoNRad represents the multi-physics, multi-scale probabilistic simulation based on physical models, while the sensor updates include the optical power, temperature, pressure, quality factor, the frequency of the QTF, and the humidity of the sample. Finally, the DT uses those sensor updates together with *ϵ* and *γ*, computed by CoNRad, to predict a photoacoustic signal U_calc_ for a random analyte concentration *c*_*CH4*_. The DT calculates a theoretical QEPAS signal $U_{\mathrm{calc}}$ for each measurement point, compares this to the measured amplitude $U_{\mathrm{meas}}$ at the set analyte concentration ${\left({\mathrm{CH}}_{4}\right)}_{\mathrm{set}}$ and then outputs the predicted methane concentration as:

(2) $${\left({\mathrm{CH}}_{4}\right)}_{\mathrm{pred}}=\frac{U_{\mathrm{meas}}}{U_{\mathrm{calc}}}\;{\left({\mathrm{CH}}_{4}\right)}_{\mathrm{set}}$$

Since the DT is based on the physical principles of photoacoustics, this approach is applicable to different analytes and holds valid over a broad range of environmental changes. Additionally, the contribution of individual physical phenomena, such as acoustic, spectral and relaxational influences can be assessed separately, thus increasing the understanding of photoacoustic sensors. As a major benefit of the DT, its utilization eliminates the need for calibration towards changing gas compositions in terms of the presented sensor. Thus, a single point calibration in any known gas matrix with any environmental parameters is sufficient. However, Eq. 2 is only applicable if the on-peak measurements show a linear trend with the analyte concentration, which is however given by PA theory for sufficiently low absorption coefficients of the analyte.

### 3.1 Experimental setup and operating concentration range

**Fig. 1:**
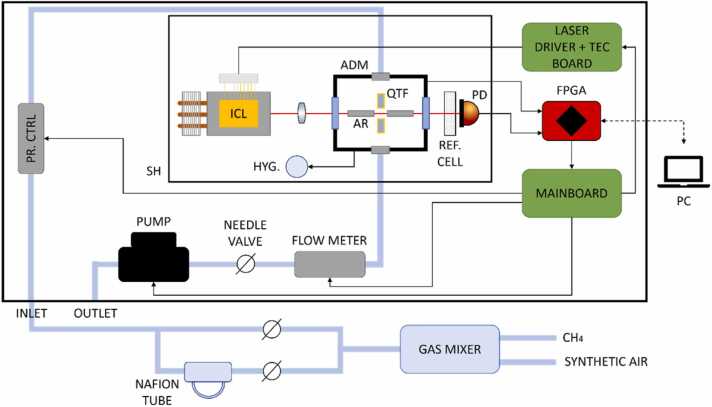
Schematic of the experimental apparatus. Black arrows represent electronic connections, dashed arrows represent USB connections, and pale blue bold lines represent the tubes used for gas supply. Finally, the aluminum box borders are represented as a bold blue rectangle. SH – Sensor head, PR. CTRL – Pressure controller, HYG. – Hygrometer, ICL – Interband Cascade Laser, QTF – Quartz tuning fork, AR – Acoustic resonator, PD – Photodiode, ADM – Acoustic detection module. (For interpretation of the references to color in this figure legend, the reader is referred to the web version of this article.)

Fig. 1 shows the architecture of the QEPAS sensor employed to retrieve methane concentration within a synthetic air matrix, consisting of 80 % nitrogen and 20 % oxygen, at different humidity levels. The apparatus is enclosed in a portable 50×50×20 cm aluminum box.

The sensor optical components are placed within the sensor head (SH), a stainless-steel box preventing the system’s misalignments and increasing the sensor’s compactness and ruggedness. A DFB-ICL (Thorlabs ID3345HHLH–A) with central emission wavelength of 3.345 µm (2989 cm^−1^) and peak power of ∼17.4 mW at T = 15 °C is used as a light source for the QEPAS sensor. The DFB-ICL is placed inside the SH and mounted on an air-cooled heat sink. It was driven by means of a custom printed circuit board (PCB), also including a thermoelectric cooler (TEC) driver chip (Thorlabs MTD1020T) to set the laser operating temperature. The PCB is connected to a RedPitaya STEMlab 125–14 evaluation board through a dedicated mainboard, and the output signals are fed to a personal computer to be acquired by means of a custom LabVIEW-based software. The laser beam is focused by means of a CaF_2_ lens with a 2–5 µm anti-reflection coating, having a focal length f = 40 mm (Thorlabs LB5864–E), within an acoustic detection module (ADM). Then, the laser beam exiting the ADM passes through a reference cell containing a certified 0.5 % CH_4_:N_2_ mixture and is collected by a photodiode (Thorlabs PDA07P2). The ADM (Thorlabs ADM01) consists of a vacuum-tight gas cell, mounting two ZnSe windows with 2–13 µm AR coating (Thorlabs WG70530-E4) and a pair of connectors for gas inlet and outlet. Inside the ADM is accommodated the QEPAS spectrophone, consisting of a custom T-shaped quartz tuning fork (QTF) and a pair of resonator tubes. The QTF is characterized by a resonance frequency of *f*_0_ = 12,458 Hz and a quality factor of *Q* = 15,600 at an operating pressure of 400 Torr [49]. The piezoelectric current generated by the QTF is converted into a voltage signal by means of a transimpedance amplifier with a 10 MΩ feedback resistor. The voltage signals are fed to the FPGA and then acquired by means of the LabVIEW-based software. The absolute humidity and the temperature inside the ADM were monitored throughout all the measurements by means of a hygrometer (iST HYT 939). The employed sensor is characterized by a humidity operating range from 0 % RH to 100 % RH and a temperature operating range from 0 °C to 60 °C, providing an absolute accuracy of ± 1.8 % RH and ± 0.2 °C. The QEPAS measurements were performed in 2 f-wavelength modulation (2 f-WM), i.e., modulating the laser at half the QTF resonance frequency and demodulating the response signal at its resonance frequency. A sinusoidal dither is used to modulate the laser source at a fixed current (“on-peak mode”). Alternatively, a slow ramp is superimposed to the fast modulation and used to scan across the laser dynamic range (“spectral scan mode”). The QTF signal is then demodulated by means of a LabVIEW-based dual phase digital lock-in amplifier, with maximum input voltage of 1 V. The lock-in integration time was set to 125 ms for all the performed measurements. Both the modulation and demodulation processes are managed by the FPGA, acquiring both the in-phase signal and the quadrature (or magnitude) signal. In the case of on-peak measurements for trace concentrations, the 3 f-signal generated by the photodiode placed beyond the ADM and the CH_4_ reference cell can be optionally used as an error signal to compensate possible temperature drifts of the laser source.

Two gas cylinders with certified CH_4_ concentration of 500 ppm and 2.177 %, respectively, in synthetic air and a gas cylinder containing synthetic air were used to generate the gas samples to be analyzed. The cylinders were provided with a 1 % expanded uncertainty on nominal concentrations. A gas mixer (MCQ Instruments GB-100) was employed to select the methane dilution ratio in synthetic air and to set the flow rate in the gas line. The gas mixer is characterized by an accuracy of 1 % of the setpoint, for each channel. The output of the gas mixer was downstream connected to a humidifier (PermSelect PDMSXA-1000) to set the in-line H_2_O concentration by varying the aperture of two needle valves, as depicted in Fig. 1, from 0.3 % up to 2 % of absolute humidity. A pressure controller (ALICAT EPC-15PSIAP01-BM0P), a flow meter (ALICAT BC-C1000), a needle valve, and a diaphragm pump (Thomas 1420VR 24 V) were embedded in the sensor box. These devices were employed to set the operating pressure and monitor the flow rate in the gas line: all the measurements were performed at 400 Torr and 50 sccm, respectively.

### 3.2 Target features selection

**Fig. 2:**
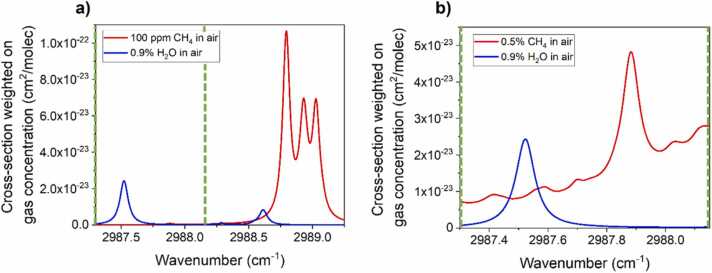
HITRAN simulation at P = 400 Torr of the spectral regions selected to acquire QEPAS spectra of gas samples with (a) low CH_4_ concentration (25–200 ppm), and (b) high CH_4_ concentration (1100–11,000 ppm, 0.11–1.10 %). The absorption spectra of CH_4_ (red lines) are simulated at concentrations representative for the concentration range, while the absorption spectra of H_2_O (blue line) are simulated at a typical atmospheric concentration. Green dashed lines in panel (a) point out the spectral region shown in panel (b).

Due to the limitation on the voltage input of the employed digital lock-in amplifier, two different concentration ranges were selected to calibrate the QEPAS sensor and perform both the DT and PLSR analysis. The first range spans from 25 ppm to 200 ppm of CH_4_ and is labelled hereafter as “low CH_4_ concentration range”, while the second range spans from 1100 ppm to 11,000 ppm (0.11–1.10 %) and is labelled hereafter as “high CH_4_ concentration range”. Thus, two spectral regions were selected within the DFB-ICL current dynamic range and simulated by using the HITRAN database [50], as reported in Fig. 2a-b together with the absorption spectrum of H_2_O at typical atmospheric concentration of 0.9 %.

The methane triplet located between 2988.50 cm^−1^ and 2989.25 cm^−1^ (Fig. 2a) was targeted to detect CH_4_ within the “low concentration range”. These features exhibit an absorption cross section weighted with CH_4_ concentration in the order of ∼10^−22^ cm^2^/molecule (see Fig. 2a). Moving to the CH_4_ detection at percentage level the use of these features would have led to two different issues, related to: i) nonlinearities in Lambert-Beer absorption; ii) lock-in signal saturation. Therefore, an absorption feature located at 2987.87 cm^−1^ was employed to target the “high CH_4_ concentration range” (Fig. 2b), since at percentage level it provided an absorption cross section weighted with CH_4_ concentration comparable to that estimated in the ppm range (Fig. 2a).

### 3.3 QEPAS sensor calibration

The gas sensor was calibrated for both the low CH_4_ concentration range and for the high CH_4_ concentration range, respectively, employing the same procedure for both data acquisition and data analysis.

The measurements were performed setting the H_2_O level in the samples and varying the CH_4_ concentration in the investigated range. The water vapor concentration was acquired by means of the hygrometer housed inside the ADM (see Fig. 1). The RH value was then converted into an absolute H_2_O concentration, with known temperature and pressure inside the cell. Once set the H_2_O concentration, two subsequent acquisition steps were performed for each CH_4_ concentration. First, the QEPAS spectrum of the CH_4_-H_2_O mixture in air was acquired operating the sensor in spectral scan mode. Then, the peak value of the selected CH_4_ absorption feature was measured operating the sensor in on-peak mode. The QEPAS spectra have been employed as dataset for the PLSR analysis, while the on-peak acquisitions have been fed to the DT analysis.

### 3.4 Low CH_4_ concentration range

The performances of the QEPAS sensor when targeting CH_4_-H_2_O mixtures with low CH_4_ concentration were evaluated analyzing 60 gas samples with different composition. Six CH_4_ concentrations, from 25 ppm up to 200 ppm, and ten H_2_O concentrations, from 0.25 % up to 1.90 % were mixed in the samples starting from the gas cylinder with certified concentrations and diluted in synthetic air.

**Fig. 3:**
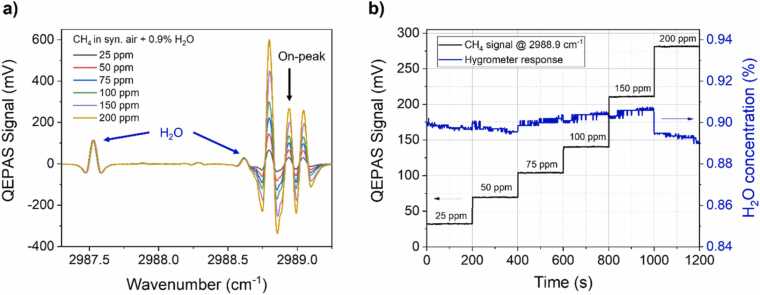
(a) 2 f-QEPAS spectral scans acquired in the low CH_4_ concentration range with a H_2_O concentration of 0.9 %. Blue arrows point towards H_2_O absorption features, black arrow points towards the CH_4_ feature used for on-peak measurements. (b) Stepwise representation of CH_4_ on-peak measurements (black line) and corresponding H_2_O concentration (blue line) calculated using the capacitive hygrometer. (For interpretation of the references to color in this figure legend, the reader is referred to the web version of this article.)

The QEPAS spectra acquired at different CH_4_ concentrations with a H_2_O level of 0.9 % are shown in Fig. 3a as representatives. Each spectrum was acquired by means of a single sweep and consists of 537 data points with a spectral resolution of ∼0.0036 cm^−1^ and total acquisition time was ∼4 min. Such a wide scan allowed the detection of both the H_2_O spectral features falling within the laser dynamic range as well as the CH_4_ triplet.

The collected QEPAS spectral scans resemble the shape of the 2nd derivative of the HITRAN simulation shown in Fig. 2a. The methane triplet can be clearly observed, being characterized by three well separated peaks whose intensities scales proportionally to the CH_4_ concentration. Two H_2_O absorption features can be observed: one at ∼2987.55 cm^−1^ well separated from the triplet, and one at ∼2988.60 cm^−1^ partially merged to the first CH_4_ peak. The detection phase for lock-in acquisition was set to the one maximizing the in-phase signal generated by the central peak of CH_4_ triplet at the lowest water concentration and was kept fixed for all the measurements.

After each spectral scan, the sensor was operated in on-peak mode, to acquire the data for DT analysis. To perform the analysis in the low CH_4_ concentration range, the QEPAS peak located at 2988.93 cm^−1^ was preferred to the adjacent and more intense one located at 2988.80 cm^−1^ to avoid any influence from the nearby water peak at ∼2988.60 cm^−1^. Each QEPAS peak signal was acquired for 200 s before changing the sample composition in the gas line, to ensure that no signal drift occurs in the sensor. The average value was considered as reference value for DT analysis. The driving current of the DFB-ICL was locked to the selected CH4 peak by means of the photodiode signal demodulated at the third harmonic, and employed as error signal [51]. The QEPAS peak signals acquired at different CH_4_ concentrations with a nominal H_2_O level of 0.9 % are shown in Fig. 3b as representatives. The water vapor monitoring acquired by the hygrometer is also reported in the same figure. During acquisitions at a fixed water vapor (Fig. 3b), small fluctuations around the mean value were observed. This effect occurs because the water vapor level is slightly influenced by the operating conditions: the valve opening dynamics of the mixer channels operates on a different time scale with respect to the passive humidifier, which reacts much slowly. Nevertheless, the measured relative variation around the mean value was within 3 %, for all the samples: this fluctuation leads to a negligible influence on CH_4_ QEPAS signal. Indeed, the relative fluctuations of the CH_4_ signal was below 2 %.

**Fig. 4:**
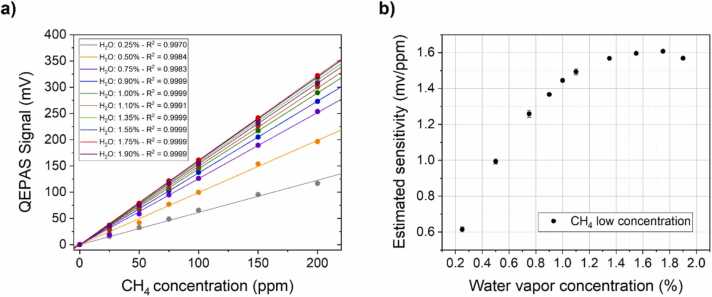
(a) QEPAS peak signal of CH_4_ in the low concentration range corresponding to the absorption feature at 2898.95 cm^−1^, for each investigate humidity level (circular dots). A linear fit is superimposed to each measurement set (solid lines), and the corresponding value of R^2^ is reported in the graph’s legend. (b) QEPAS sensor sensitivities estimated by the best linear fit as a function of H_2_O concentration.

To point out the effects of H_2_O on CH_4_ photoacoustic response, the QEPAS peak signals were plotted in Fig. 4a as a function of the CH_4_ concentration, for each humidity level.

The linear response of the sensor is verified in the investigated concentration range for each humidity level. As expected, the sensitivity the CH_4_ QEPAS changes when the H_2_O concentration is varied (Fig. 4b). For H_2_O concentrations up to 1.0 % the sensitivity increases accordingly, while at higher H_2_O concentrations the sensor’s response reaches a plateau with a slight decrease at humidity level beyond 1.8 %.

### 3.5 High CH_4_ concentration range

The performances of the QEPAS sensor when targeting CH_4_-H_2_O mixtures with high CH_4_ concentration were evaluated analyzing 60 gas samples with different composition. Six CH_4_ concentrations, from 0.11 % up to 1.10 %, and ten H_2_O concentrations, from 0.20 % up to 1.95 % were mixed in the samples starting from the gas cylinder with certified concentrations to be diluted in synthetic air.

**Fig. 5:**
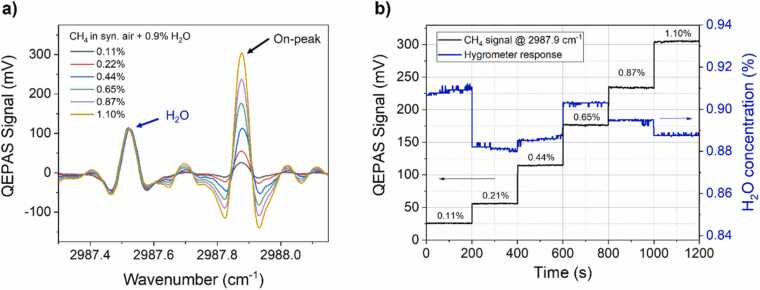
(a) 2 f-QEPAS spectral scans acquired in the high CH_4_ concentration range with a H_2_O concentration of 0.9 %. Blue arrow points towards H_2_O absorption feature. (b) Stepwise representation of CH_4_ on-peak measurements (black line) and corresponding H_2_O concentration (blue line).

The QEPAS spectrum of each gas sample was acquired operating the sensor in spectral scan mode. The QEPAS spectra acquired at different CH_4_ concentrations with a H_2_O level of 0.9 % are shown in Fig. 5a as representatives. Each spectrum was acquired by means of a single sweep and consisted in 237 data points with a spectral resolution of ∼0.0036 cm^−1^ and total acquisition time was ∼2 min.

The methane absorption feature located at 2987.87 cm^−1^, as well as some other minor CH_4_ features, can be observed in the full spectral scan. The H_2_O absorption feature located at 2987.55 cm^−1^ can be also observed in the collected scans. Differently from the previous case, a minor interference among CH_4_ and H_2_O absorptions is observed. The strongest CH_4_ feature at 2987.87 cm^−1^ was used for the on-peak measurements. The detection phase for lock-in acquisition was set to the one maximizing the in-phase signal generated by the selected CH_4_ absorption feature and kept fixed for all the measurements.

Analogously to the measurements in low CH_4_ concentration range, after each spectral scan the QEPAS peak signal was acquired for 200 s. The QEPAS peak signals acquired at different CH_4_ concentrations with a nominal H_2_O level of 0.9 % are shown in Fig. 5b, together with the H_2_O concentration monitoring provided by the capacitive hygrometer. As for the measurements at low CH_4_ concentrations, relative fluctuations < 3 % around the mean value were observed during the measurement sessions at fixed water concentrations, leading to a negligible effect on CH_4_ peak signal amplitude which exhibited relative fluctuation < 2 % for all generated gas samples.

**Fig. 6:**
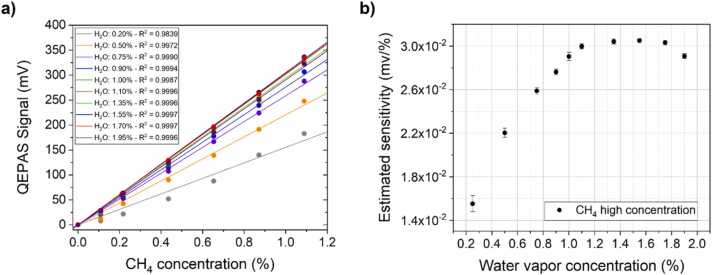
QEPAS peak signal of CH_4_ in the high concentration range corresponding to the absorption feature at 2897.87 cm^−1^, for each investigate humidity level (circular dots). A linear fit is superimposed to each measurement set (solid lines), and the corresponding value of R^2^ is reported in graph’s legend. (b) QEPAS sensor sensitivities estimated by the best linear fit as a function of H_2_O concentration.

The QEPAS peak signals as a function of the CH_4_ concentration for each humidity level were plotted in Fig. 6a.

The linear response of the sensor is verified also in the high CH_4_ concentration range, for each humidity level. The sensitivity of the CH_4_ QEPAS sensor depends on the humidity level (Fig. 6b), exhibiting a trend similar to the one observed for the low CH_4_ concentrations (Fig. 4b). Up to H_2_O concentrations of 1.0 %, the CH_4_ response increases accordingly, while at higher H_2_O concentrations the sensor’s response reaches a plateau with a slight decrease at humidity level beyond 1.80 %.

### 3.6 Data analysis and concentration retrieval

The information acquired from spectral scans and on-peak measurements were used to perform PLSR and DT analysis, respectively, aiming for filtering out the effects of variable water vapor on the CH_4_ sensitivity and returning an accurate prediction of CH_4_ concentration.

### 3.7 DT algorithm optimization

The core of the DT is the algorithm CoNRad presented in the previous section, which allows the user to calculate the expected relaxational efficiency, based on the measurement conditions. The on-peak QEPAS amplitude as well as its phase were included into the evaluation process since the phase also contains information about cross-influences. In cases where the measured amplitude is well reproduced by the theoretical one, but significant discrepancies occur between measured and theoretical phase shift, it must be assumed that the physical model of the DT lacks completeness, e.g. not considering relevant energy transitions, or assuming wrong transition rates. A complete relaxational diagram including the corresponding transition rates for mid-infrared methane detection in humidified air was already presented in Ref.[48]. The relaxations considered for the investigated samples as well as the reaction rate employed in the CoNRad algorithm are reported in Table S1 of supplementary file.

### 3.8 PLSR algorithm optimization

PLSR data analysis was performed independently for each concentration range, using the same algorithm. The predictors matrix ***X*** was assembled starting from the acquired spectral scans, while the response matrix ***Y*** contains the corresponding nominal concentrations. Both in-phase and quadrature spectra acquired from the lock-in amplifier were employed in the analysis to account for the phase shift on CH_4_ QEPAS signals induced by different H_2_O concentrations. The spectral acquisitions shown in Figs. 3a and 5a were employed for PLSR analysis, covering the spectral range from 2989.3 cm^−1^ - 2987.3 cm^−1^ and 2988.2 cm^−1^ - 2987.3 cm^−1^ for low and high CH_4_ concentration, respectively. PLSR was performed in machine learning-like approach, splitting the dataset into a calibration set and a test set. To perform a consistent comparison with the DT analysis each sample was tested independently, thus with a single test sample and with 59 calibration samples.

**Fig. 7:**
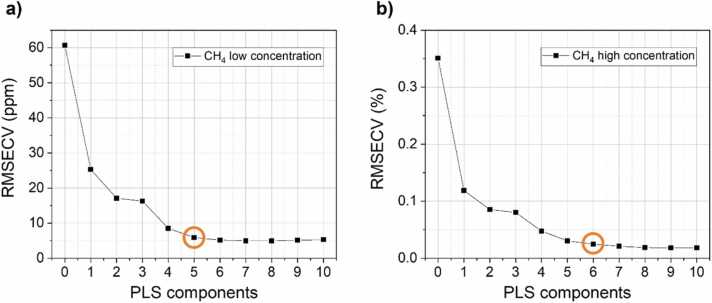
RMSECV as a function of the number of PLS components for (a) low CH_4_ concentrations and (b) high CH_4_ concentrations. Orange circles point out the number of LVs selected for the analysis. (For interpretation of the references to color in this figure legend, the reader is referred to the web version of this article.)

A 10-fold cross-validation (CV) analysis was performed prior to the test step to determine the optimal number of LVs for each concentration range. The LVs evaluation was operated on the full measurement dataset. The CV errors, expressed as root-mean-square error (RMSECV), calculated for the two concentration ranges are shown in Fig. 7a-b.

Calibration dataset for low CH_4_ concentrations shows the decrease of RMSECV up to 5 PLS components, namely LVs, while the error values corresponding to a larger number of components are characterized by negligible variations. Conversely, the calibration dataset for high CH_4_ concentrations shows a significant RMSECV decrease up to 6 PLS components. PLSR is not meant to be interpreted as an explainable AI [52], but it is possible to correlate the LVs with independent, physically relevant parameters contributing to the spectra [42]. In this case, it would be possible to assign a LV to: i) CH_4_ concentration; ii) H_2_O concentration; iii) the photoacoustic relaxation rate of CH_4_ trough H_2_O; iv) phase of the acquired signal for each water concentration; and v) resonance properties of the QEPAS spectrophone affected by variations of fluid dynamics properties in the gas samples. The additional components observed at high CH_4_ concentration can be ascribed to the self-relaxation of CH_4_, which can be assumed negligible at low concentrations [26].

### 3.9 Results comparison

**Fig. 8:**
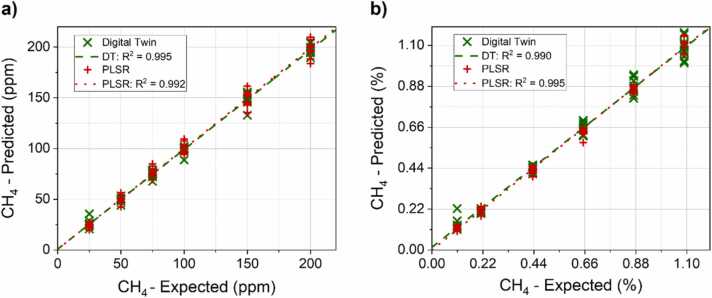
Comparison between the DT (green ×) and PLSR (red +) results for (a) low CH_4_ concentrations and (b) high CH_4_ concentrations. Methane concentrations retrieved using the algorithms are plotted against the expected, nominal, concentrations in the samples. A linear fit is superimposed to the both the DT data (green dashed line) and PLSR data (red dotted line). The corresponding R^2^ values are reported in graph’s legend. (For interpretation of the references to color in this figure legend, the reader is referred to the web version of this article.)

All the acquired spectra were tested employing the algorithms configurations presented in the previous paragraphs. The CH_4_ concentrations retrieved employing DT and PLSR analysis targeting the low and high CH_4_ concentration range are shown in Fig. 8a-b, respectively.

The achieved results reveal a linear trend of the predicted values versus the expected values for both the concentration ranges and both the analysis methods, as demonstrated by the superimposed best linear fit, whose calculated parameters are reported in Table 1.

**Table 1:** Results of best linear fit superimposed to the predicted versus expected concentration graphs.

	Low CH_4_ concentration range			High CH_4_ concentration range		
	R^2^	Slope	Intercept (ppm)	R^2^	Slope	Intercept (%)
DT	0.995	0.981 ± 0.009	0.928 ± 1.038	0.990	0.983 ± 0.013	0.015 ± 0.009
PLSR	0.992	0.992 ± 0.012	0.725 ± 1.372	0.996	1.001 ± 0.009	0.001 ± 0.006

The R^2^ values are ≥ 0.990 and the calculated slopes approach the ideal value of 1. Both the analysis techniques point out a negligible intercept within the error limits, < 1σ for all the datasets except for DT at high concentrations (<2σ).

The average relative error of prediction (AREP) as well as the mean absolute deviation from the expected values were used to evaluate the accuracy of the predicted concentrations. These values, corresponding to each data analysis technique and each concentration range, are shown in Table 2.

**Table 2:** Results comparison between DT and PLSR analysis for both low and high CH_4_ concentration range in terms of calculated AREP and absolute deviation.

	DT – AREP (rel.%)	PLSR – AREP (rel.%)	DT - Abs. dev. (ppm)	PLSR - Abs. dev. (ppm)
Low CH_4_ range	3.8 %	4.6 %	2.9	3.9
High CH_4_ range	7.6 %	4.9 %	248	179

In the low CH_4_ concentration range, DT results are slightly better than PLSR predictions providing lower discrepancies and thus higher accuracies. Conversely, in the high CH_4_ concentration range, PLSR shows a higher accuracy compared to DT. Comparing the two concentration ranges, the high concentration one shows lower accuracy for both analysis methods, with a slight degradation (from 4.6 % to 4.9 %) for PLSR and a more significant one (from 3.8 % to 7.6 %) for DT. The one-to-one comparison of the retrieved results is reported in Table S2 and Table S3 of supplementary files. The achieved results show variable discrepancies between the nominal and predicted concentrations. In particular, the test samples characterized by lowest CH_4_ concentrations in both concentration ranges (25 ppm and 0.11 %) as well as the test samples characterized by the lowest H_2_O level (0.20 % and 0.25 %) pointed out the lowest relative accuracy in the dataset.

**Fig. 9:**
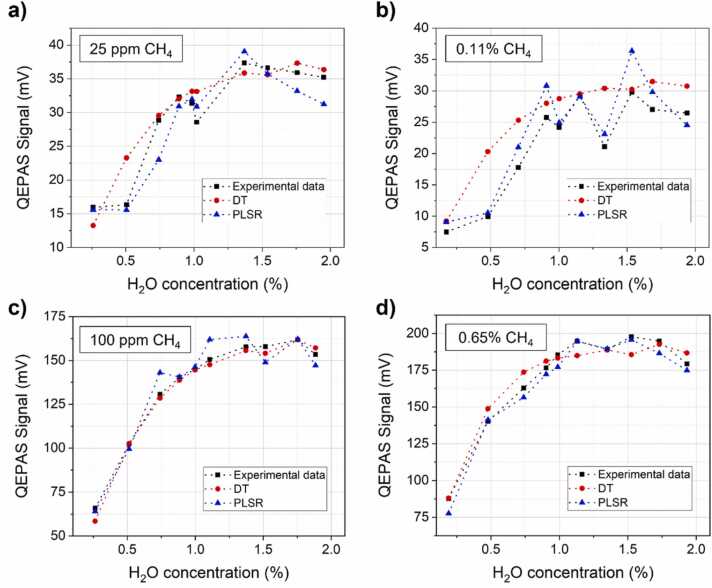
Comparison among the QEPAS signal acquired experimentally (black squares), calculated theoretically using DT (red dots) and regressed from PLSR predicted concentrations (blue triangles) for the set characterized by CH_4_ concentration of (a) 25 ppm; (b) 0.11 %; (c) 100 ppm; (d) 0.65 %. (For interpretation of the references to color in this figure legend, the reader is referred to the web version of this article.)

The interpretation of the obtained results can be effectively pursued in the perspective of the intrinsic characteristics and differences between the two analysis approaches. If the components of the gas matrix are known and their physical properties in terms of relaxation pathways fully and accurately determined, DT analysis is expected to be more accurate than PLSR, being supported by a systematic computation of all the contributions to the QEPAS signal generation rather than the statistical interpretation of the spectra. In addition, DT analysis can be easily implemented for real-time conversion of QEPAS signal into target gas concentration. Moreover, relying on a full characterization of energy dynamics, the algorithm can be easily adapted to target another gas species or to address the effect of a matrix variation, once provided collisional partners and related energy levels. The main drawback of DT approach is that it requires a complete knowledge of all the involved physical phenomena as well as a full control of all the parameters characterizing the complex gas mixtures. Instrumental parameters, high inaccuracy of instruments when operating close to their limit (as for the generation of the lowest concentrations, i.e., the 25 ppm and 0.11 % CH_4_-mixtures), variations of relaxation dynamics when a large number of target molecules are involved (potentially responsible for the low accuracy of DT in the high CH_4_ concentrations range) are hard to be modelled. Conversely, PLSR points out a higher versatility characterized by stable performances which can be easily generalized to different datasets. Due to the multivariate nature of the analysis, PLSR can deal with overlapping features and be less prone to inaccuracy due to external instruments, i.e., the hygrometer, when the information is included in the spectra. The main drawback is that the results obtained for a sensor cannot be generalized for another sensor and time-consuming acquisitions to build a training dataset are always mandatory. To visualize these effects, the collected QEPAS peak signals are compared with the signals theoretically calculated by the DT. In addition, we included the QEPAS signal regressed from the predicted PLSR concentrations exploiting the linear response of the sensor, and the results are shown in Fig. 9. These signals are close but not coincident to the peak signals acquired in the spectral scans like the ones showed in Fig. 5, as the PLSR analysis account for all the data to retrieve the concentrations.

The data reported in Figs. 9a and 9b were acquired setting the same experimental parameters in the gas line (pressure, flow rate, temperature, and gas mixer apertures), changing the starting certified concentration. The same applies to the data reported in Figs. 9c and 9d. The first pair of graphs exhibits the higher signal fluctuations ascribed to instrumental accuracy, while the second pair points out the excellent theoretical modelling at low concentrations compared to a less accurate one in the high concentrations range.

The possibility to model sensor’s behaviors that are repeatable and related to minor but physically relevant phenomena can be an advantage of statistically based approach like PLSR, however the occurrence of overfitting as well as systematic errors should be carefully evaluated. Conversely, the advantages of theoretically based approaches like DT relies on the possibility to simulate the sensor behavior upstream filtering the possible experimental fluctuations. Aiming to develop a gas sensor for on field operation this may represent a disadvantage or an advantage as moving outside the controlled laboratory environment it is easy to run into unpredictable and uncontrolled fluctuations affecting sensor’s calibration.

The effects of the measurements set characterized by the higher discrepancies on the overall accuracy can be evaluated in terms of relative and absolute accuracy. Removing the measurements with 25 ppm of CH_4_ the DT-AREP reduces from 3.8 % to 2.9 % while its absolute deviation increases from 2.9 ppm to 3.0 ppm. In turn the PLSR-AREP reduces from 4.6 % to 4.2 % while its absolute deviation increases from 3.9 ppm to 4.4 ppm. Removing the measurements with 0.11 % of CH_4_ the DT-AREP reduces from 7.6 % to 3.5 % while its absolute deviation reduces from 248 ppm to 237 ppm. In turn the PLSR-AREP reduces from 4.9 % to 3.5 % while its absolute deviation increases from 179 ppm to 189 ppm.

## 4 Conclusions

In this work, a comparative investigation upon two different data analysis techniques used to compensate the effects of molecular relaxation in photoacoustic spectroscopy is presented. The first one was based on PLSR analysis while the second one was based on the DT representation of the experimental system. To address this task, a dedicated QEPAS sensor was developed and embedded in a shoe-size box containing the sensor head, the electronic instrumentation, and the gas line management devices. For this case study, CH_4_ was selected as target molecule and the effects of variable H_2_O concentration (from 0.3 % up to 2.0 %) within a synthetic air matrix were investigated in the spectral range around 3.345 µm. Two CH_4_ concentration ranges were explored, one in the ppm range and one in the percent range, to account for different applications. PLSR-based approach relies on the statistical evaluation of the collected spectral scans to model the cross-correlation among the analytes in the gas sample and retrieve the target concentration. DT-based approach relies on an algorithmic approach to compute the whole relaxation dynamics occurring in the gas sample by exploiting the collected on-peak target signal. Despite of the strong relaxation effects induced by the different humidity levels in the samples, both analysis tools were able to return target concentrations with average accuracy ∼95 %. In the low concentration range, DT performed better compared to PLSR benefitting of its strong theoretical background. In the high concentration range, an opposite situation was observed that can be ascribed to a partial spectral interference among H_2_O and CH_4_. Further developments of both analysis algorithms will be focused on improving the achieved accuracy as well as on testing more complex mixtures, in terms of number of analytes as well as spectral and non-spectral cross-interference effects.

## Declaration of Competing Interest

The authors declare the following financial interests/personal relationships which may be considered as potential competing interests: Angelo Sampaolo reports was provided by Polytechnic University of Bari.

## Data Availability

Data will be made available on request.
