# Supplementary material for: Multivariate analysis and digital twin modelling: Alternative approaches to evaluate molecular relaxation in photoacoustic spectroscopy
Source: Photoacoustics. 2023 Oct 9;33:100564. doi: 10.1016/j.pacs.2023.100564 (PMC10658604; doi:10.1016/j.pacs.2023.100564)
Supplement: Supplementary file 1 — Supplementary material [file mmc1.docx]

**Table S1:** Summary of all collision-based energy transitions (reactions) and respective reaction rate k for the complete de-excitation process during photoacoustic methane detection with regards to gas matrices containing nitrogen, oxygen and water.

|  | **Reaction** | **Reaction rate *k* in s^-1^ atm^-1^**  **used literature** | |
| --- | --- | --- | --- |
| $\left( 1 \right)$ | $\mathrm{CH}_{4}\left( \nu_{s1} \right)+M \underset{\to}{k_{1}^{M}}\mathrm{CH}_{4}\left( {2\nu}_{b} \right) +M$ | $k_{1}^{\mathrm{CH}_{4}} = 2.1\cdot{10}^{8}$ | $2.1\cdot{10}^{8}$ [1] |
|  |  | $k_{1}^{N_{2}} = 4.6\cdot{10}^{8}$ | $4.6\cdot{10}^{8}$ [2] |
|  |  | $k_{1}^{H_{2}O}= 1\cdot{10}^{8}$ | $/$ |
|  |  | $k_{1}^{O_{2}} = 4.6\cdot{10}^{8}$ | $4.6\cdot{10}^{8}$ [2] |
| $\left( 2 \right)$ | $\mathrm{CH}_{4}\left( {2\nu}_{b} \right)+\mathrm{CH}_{4} \underset{\to}{k_{2}} \mathrm{CH}_{4}\left( \nu_{b} \right)+\mathrm{CH}_{4}\left( \nu_{b} \right)$ | $k_{2} = 5\cdot{10}^{8}$ | $5.5\cdot{10}^{8}$ [3] |
| $\left( 3 \right)$ | $\mathrm{CH}_{4}\left( {2\nu}_{b} \right) +H_{2}O \underset{\to}{k_{3}} \mathrm{CH}_{4}\left( \nu_{b} \right) +H_{2}O\left( \nu_{2} \right)$ | $k_{3}= 1\cdot{10}^{8}$ | $/$ |
| $\left( 4 \right)$ | $\mathrm{CH}_{4}\left( {2\nu}_{b} \right)+O_{2} \underset{\to}{k_{4}} \mathrm{CH}_{4}\left( \nu_{b} \right)+O_{2}\left( \nu\right)$ | $k_{4} = 1\cdot{10}^{7}$ | $6.6\cdot{10}^{6}$ [3] |
| $\left( 5 \right)$ | $\mathrm{CH}_{4}\left( {2\nu}_{b} \right)+M \underset{\to}{k_{5}^{M}} \mathrm{CH}_{4}\left( \nu_{b} \right)+M$ | $k_{5}^{\mathrm{CH}_{4}}=1.6\cdot{10}^{6}$ | $1.6\cdot{10}^{6}$ [4] |
|  |  | $k_{5}^{N_{2}} =2\cdot{10}^{5}$ | $1.6\cdot{10}^{5}$ [4] |
|  |  | $k_{5}^{H_{2}O}=2.4\cdot{10}^{5}$ | $/$ |
|  |  | $k_{5}^{O_{2}} =2.6\cdot{10}^{5}$ | $/$ |
| $\left( 6 \right)$ | $\mathrm{CH}_{4}\left( \nu_{b} \right)+H_{2}O \underset{\to}{k_{6}} \mathrm{CH}_{4}+H_{2}O\left( \nu_{2} \right)$ | ${k_{6}}=7\cdot{10}^{7}$ | $/$ |
| $\left( 7 \right)$ | $\mathrm{CH}_{4}\left( \nu_{b} \right)+O_{2} \underset{\to}{k_{7}} \mathrm{CH}_{4}+O_{2}\left( \nu\right)$ | ${k_{7}}=2\cdot{10}^{6}$ | $3.3\cdot{10}^{6}$ [3] |
| $\left( 8 \right)$ | $O_{2}\left( \nu\right)+H_{2}O \underset{\to}{k_{8}} O_{2}+H_{2}O\left( \nu_{2} \right)$ | ${k_{8}}=2.2\cdot{10}^{7}$ | $2.2\cdot{10}^{8}$ [5] |
| $\left( 9 \right)$ | $O_{2}\left( \nu\right)+\mathrm{CH}_{4} \underset{\to}{k_{9}} O_{2}+\mathrm{CH}_{4}\left( \nu_{b} \right)$ | $k_{9} =3.3\cdot{10}^{7}$ | $3.3\cdot{10}^{7}$ [3] |
| $\left( 10 \right)$ | $H_{2}O\left( \nu_{2} \right)+\mathrm{CH}_{4} \underset{\to}{k_{10}} H_{2}O+\mathrm{CH}_{4}\left( \nu_{b} \right)$ | $k_{10} =2\cdot{10}^{7}$ | $/$ |
| $\left( 11 \right)$ | $H_{2}O\left( \nu_{2} \right)+O_{2} \underset{\to}{k_{11}} H_{2}O+O_{2}\left( \nu\right)$ | $k_{11}=2\cdot{10}^{7}$ | $3.8\cdot{10}^{7}$ [6] |
| $\left( 12 \right)$ | $\mathrm{CH}_{4}\left( \nu_{b} \right)+M \underset{\to}{k_{12}^{M}} \mathrm{CH}_{4}+M$ | $k_{12}^{\mathrm{CH}_{4}} =8\cdot{10}^{5}$ | $8\cdot{10}^{5}$ [3] |
|  |  | $k_{12}^{N_{2}} =4.5\cdot{10}^{4}$ | $8\cdot{10}^{4}$ [4] |
|  |  | $k_{12}^{H_{2}O}=1.2\cdot{10}^{5}$ | $/$ |
|  |  | $k_{12}^{O_{2}} =7\cdot{10}^{4}$ | $1.4\cdot{10}^{5}$ [3] |
| $\left( 13 \right)$ | $H_{2}O\left( \nu_{2} \right)+M \underset{\to}{k_{14}^{M}} H_{2}O+M$ | $k_{13}^{\mathrm{CH}_{4}} =1\cdot{10}^{6}$ | $/$ |
|  |  | $k_{13}^{N_{2}} =1\cdot{10}^{7}$ | $1\cdot{10}^{6}$ [5] |
|  |  | $k_{13}^{H_{2}O}=1.2\cdot{10}^{9}$ | $1.2\cdot{10}^{9}$ [7] |
|  |  | $k_{13}^{O_{2}} =1\cdot{10}^{6}$ | $1\cdot{10}^{6}$ [5] |
|  |  |  |  |
| $\left( 14 \right)$ | $O_{2}\left( \nu\right)+M \underset{\to}{k_{13}^{M}} O_{2}+M$ | $k_{14}^{\mathrm{CH}_{4}} =1\cdot{10}^{6}$ | $1\cdot{10}^{6}$ [8] |
|  |  | $k_{14}^{N_{2}} =18$ | $18$ [9] |
|  |  | $k_{14}^{H_{2}O}=1.1\cdot{10}^{6}$ | $8\cdot{10}^{5}$ [5] |
|  |  | $k_{14}^{O_{2}} =81$ | $81$ [7] |

**Table S2:** Results of data analysis on low CH_4_ concentration range using DT and PLSR.

| Nominal H_2_O conc. (%) | Nominal CH_4_ conc. (ppm) | DT predicted CH_4_ conc. (ppm) | PLSR predicted CH_4_ conc. (ppm) | DT relative deviation (rel.%) | PLSR relative deviation (rel.%) | Highest accuracy |
| --- | --- | --- | --- | --- | --- | --- |
| 0.25 | 25.0 | 20.8 | 24.4 | 16.9 | 2.3 | PLSR |
| 0.50 | 25.0 | 35.6 | 23.8 | 42.5 | 4.7 | PLSR |
| 0.75 | 25.0 | 25.6 | 20.0 | 2.6 | 20.2 | DT |
| 0.90 | 25.0 | 24.8 | 23.9 | 0.9 | 4.4 | DT |
| 1.00 | 25.0 | 26.4 | 25.5 | 5.6 | 1.9 | PLSR |
| 1.10 | 25.0 | 29.0 | 27.0 | 15.9 | 8.0 | PLSR |
| 1.35 | 25.0 | 24.0 | 26.2 | 4.0 | 4.6 | DT |
| 1.55 | 25.0 | 24.3 | 24.4 | 2.8 | 2.5 | PLSR |
| 1.75 | 25.0 | 26.0 | 23.1 | 3.9 | 7.6 | DT |
| 1.90 | 25.0 | 25.8 | 22.2 | 3.2 | 11.3 | DT |
| 0.25 | 50.0 | 44.0 | 50.7 | 12.1 | 1.3 | PLSR |
| 0.50 | 50.0 | 50.2 | 42.6 | 0.5 | 14.8 | DT |
| 0.75 | 50.0 | 48.6 | 53.8 | 2.8 | 7.5 | DT |
| 0.90 | 50.0 | 48.7 | 48.4 | 2.7 | 3.3 | DT |
| 1.00 | 50.0 | 50.7 | 50.9 | 1.4 | 1.7 | DT |
| 1.10 | 50.0 | 50.8 | 56.3 | 1.6 | 12.7 | DT |
| 1.35 | 50.0 | 48.4 | 50.2 | 3.2 | 0.3 | PLSR |
| 1.55 | 50.0 | 47.8 | 50.8 | 4.4 | 1.7 | PLSR |
| 1.75 | 50.0 | 50.0 | 51.0 | 0.0 | 2.0 | DT |
| 1.90 | 50.0 | 53.5 | 47.1 | 6.9 | 5.8 | PLSR |
| 0.25 | 75.0 | 67.7 | 75.4 | 9.8 | 0.5 | PLSR |
| 0.50 | 75.0 | 75.6 | 69.6 | 0.8 | 7.2 | DT |
| 0.75 | 75.0 | 75.6 | 82.8 | 0.8 | 10.4 | DT |
| 0.90 | 75.0 | 75.5 | 73.5 | 0.7 | 2.0 | DT |
| 1.00 | 75.0 | 77.4 | 79.0 | 3.2 | 5.3 | DT |
| 1.10 | 75.0 | 76.3 | 84.8 | 1.8 | 13.1 | DT |
| 1.35 | 75.0 | 73.8 | 78.1 | 1.5 | 4.2 | DT |
| 1.55 | 75.0 | 72.0 | 73.9 | 4.0 | 1.4 | PLSR |
| 1.75 | 75.0 | 76.6 | 77.6 | 2.2 | 3.5 | DT |
| 1.90 | 75.0 | 78.4 | 74.3 | 4.6 | 1.0 | PLSR |
| 0.25 | 100.0 | 88.8 | 97.3 | 11.2 | 2.7 | PLSR |
| 0.50 | 100.0 | 100.4 | 97.3 | 0.4 | 2.7 | DT |
| 0.75 | 100.0 | 98.3 | 109.5 | 1.7 | 9.5 | DT |
| 0.90 | 100.0 | 98.9 | 100.2 | 1.1 | 0.2 | PLSR |
| 1.00 | 100.0 | 99.8 | 101.0 | 0.2 | 1.0 | DT |
| 1.10 | 100.0 | 98.0 | 107.5 | 2.0 | 7.5 | DT |
| 1.35 | 100.0 | 98.6 | 103.8 | 1.4 | 3.8 | DT |
| 1.55 | 100.0 | 97.5 | 94.3 | 2.5 | 5.7 | DT |
| 1.75 | 100.0 | 100.0 | 100.2 | 0.0 | 0.2 | DT |
| 1.90 | 100.0 | 102.4 | 95.9 | 2.4 | 4.1 | DT |
| 0.25 | 150.0 | 132.8 | 146.1 | 11.4 | 2.6 | PLSR |
| 0.50 | 150.0 | 153.9 | 135.2 | 2.6 | 9.9 | DT |
| 0.75 | 150.0 | 147.2 | 161.4 | 1.9 | 7.6 | DT |
| 0.90 | 150.0 | 148.6 | 144.0 | 0.9 | 4.0 | DT |
| 1.00 | 150.0 | 155.7 | 152.4 | 3.8 | 1.6 | PLSR |
| 1.10 | 150.0 | 147.5 | 155.2 | 1.6 | 3.5 | DT |
| 1.35 | 150.0 | 149.3 | 154.0 | 0.4 | 2.6 | DT |
| 1.55 | 150.0 | 145.7 | 145.8 | 2.9 | 2.8 | PLSR |
| 1.75 | 150.0 | 150.0 | 153.2 | 0.0 | 2.2 | DT |
| 1.90 | 150.0 | 153.2 | 142.8 | 2.1 | 4.8 | DT |
| 0.25 | 200.0 | 189.5 | 195.1 | 5.3 | 2.4 | PLSR |
| 0.50 | 200.0 | 194.5 | 183.9 | 2.8 | 8.1 | DT |
| 0.75 | 200.0 | 196.7 | 209.9 | 1.6 | 5.0 | DT |
| 0.90 | 200.0 | 195.9 | 201.9 | 2.0 | 0.9 | PLSR |
| 1.00 | 200.0 | 205.2 | 200.9 | 2.6 | 0.5 | PLSR |
| 1.10 | 200.0 | 194.9 | 207.1 | 2.5 | 3.6 | DT |
| 1.35 | 200.0 | 198.9 | 200.2 | 0.6 | 0.1 | PLSR |
| 1.55 | 200.0 | 194.3 | 196.9 | 2.9 | 1.6 | PLSR |
| 1.75 | 200.0 | 200.0 | 201.5 | 0.0 | 0.7 | DT |
| 1.90 | 200.0 | 204.0 | 188.5 | 2.0 | 5.7 | DT |

**Table S3** Results of data analysis on high CH_4_ concentration range using DT and PLSR.

| Nominal H_2_O conc. (%) | Nominal CH_4_ conc. (ppm) | DT predicted CH_4_ conc. (ppm) | PLSR predicted CH_4_ conc. (ppm) | DT relative deviation (rel.%) | PLSR relative deviation (rel.%) | Highest accuracy |
| --- | --- | --- | --- | --- | --- | --- |
| 0.20 | 1089 | 1337 | 1316 | 22.8 | 20.8 | PLSR |
| 0.50 | 1089 | 2227 | 1154 | 104.5 | 5.9 | PLSR |
| 0.75 | 1089 | 1551 | 1287 | 42.4 | 18.2 | PLSR |
| 0.90 | 1089 | 1186 | 1305 | 8.9 | 19.8 | DT |
| 1.00 | 1089 | 1294 | 1122 | 18.9 | 3.0 | PLSR |
| 1.10 | 1089 | 1098 | 1082 | 0.9 | 0.7 | PLSR |
| 1.35 | 1089 | 1570 | 1192 | 44.1 | 9.5 | PLSR |
| 1.55 | 1089 | 1104 | 1328 | 1.4 | 21.9 | DT |
| 1.70 | 1089 | 1269 | 1201 | 16.6 | 10.3 | PLSR |
| 1.95 | 1089 | 1265 | 1010 | 16.2 | 7.3 | PLSR |
| 0.20 | 2117 | 2000 | 1965 | 5.5 | 7.2 | DT |
| 0.50 | 2117 | 2232 | 2243 | 5.4 | 5.9 | DT |
| 0.75 | 2117 | 2095 | 2308 | 1.0 | 9.0 | DT |
| 0.90 | 2117 | 2082 | 2309 | 1.6 | 9.1 | DT |
| 1.00 | 2117 | 2062 | 2194 | 2.6 | 3.6 | DT |
| 1.10 | 2117 | 2006 | 2219 | 5.2 | 4.8 | PLSR |
| 1.35 | 2117 | 2049 | 2133 | 3.2 | 0.7 | PLSR |
| 1.55 | 2117 | 1956 | 2347 | 7.6 | 10.9 | DT |
| 1.70 | 2117 | 2110 | 2212 | 0.3 | 4.5 | DT |
| 1.95 | 2117 | 2132 | 1833 | 0.7 | 13.4 | DT |
| 0.20 | 4354 | 4190 | 3948 | 3.8 | 9.3 | DT |
| 0.50 | 4354 | 4578 | 4324 | 5.1 | 0.7 | PLSR |
| 0.75 | 4354 | 4544 | 4301 | 4.4 | 1.2 | PLSR |
| 0.90 | 4354 | 4463 | 4474 | 2.5 | 2.8 | DT |
| 1.00 | 4354 | 4424 | 4191 | 1.6 | 3.8 | DT |
| 1.10 | 4354 | 4088 | 4402 | 6.1 | 1.1 | PLSR |
| 1.35 | 4354 | 4316 | 4514 | 0.9 | 3.7 | DT |
| 1.55 | 4354 | 4120 | 4427 | 5.4 | 1.7 | PLSR |
| 1.70 | 4354 | 4333 | 4298 | 0.5 | 1.3 | DT |
| 1.95 | 4354 | 4565 | 4118 | 4.8 | 5.4 | DT |
| 0.20 | 6531 | 6556 | 5778 | 0.4 | 11.5 | DT |
| 0.50 | 6531 | 6930 | 6577 | 6.1 | 0.7 | PLSR |
| 0.75 | 6531 | 6966 | 6276 | 6.7 | 3.9 | PLSR |
| 0.90 | 6531 | 6702 | 6372 | 2.6 | 2.4 | PLSR |
| 1.00 | 6531 | 6452 | 6240 | 1.2 | 4.5 | DT |
| 1.10 | 6531 | 6200 | 6530 | 5.1 | 0.0 | PLSR |
| 1.35 | 6531 | 6515 | 6543 | 0.2 | 0.2 | PLSR |
| 1.55 | 6531 | 6130 | 6465 | 6.1 | 1.0 | PLSR |
| 1.70 | 6531 | 6459 | 6253 | 1.1 | 4.3 | DT |
| 1.95 | 6531 | 6802 | 6371 | 4.2 | 2.5 | PLSR |
| 0.20 | 8708 | 8707 | 8458 | 0.0 | 2.9 | DT |
| 0.50 | 8708 | 9428 | 8777 | 8.3 | 0.8 | PLSR |
| 0.75 | 8708 | 9336 | 8496 | 7.2 | 2.4 | PLSR |
| 0.90 | 8708 | 9106 | 8795 | 4.6 | 1.0 | PLSR |
| 1.00 | 8708 | 8626 | 8424 | 0.9 | 3.3 | DT |
| 1.10 | 8708 | 8313 | 8848 | 4.5 | 1.6 | PLSR |
| 1.35 | 8708 | 8621 | 8727 | 1.0 | 0.2 | PLSR |
| 1.55 | 8708 | 8157 | 8621 | 6.3 | 1.0 | PLSR |
| 1.70 | 8708 | 8682 | 8423 | 0.3 | 3.3 | DT |
| 1.95 | 8708 | 8813 | 8540 | 1.2 | 1.9 | DT |
| 0.20 | 10890 | 11140 | 11100 | 2.3 | 1.9 | PLSR |
| 0.50 | 10890 | 11693 | 10886 | 7.4 | 0.0 | PLSR |
| 0.75 | 10890 | 11588 | 11114 | 6.4 | 2.1 | PLSR |
| 0.90 | 10890 | 11050 | 11500 | 1.5 | 5.6 | DT |
| 1.00 | 10890 | 10444 | 11171 | 4.1 | 2.6 | PLSR |
| 1.10 | 10890 | 10154 | 11122 | 6.8 | 2.1 | PLSR |
| 1.35 | 10890 | 10717 | 11518 | 1.6 | 5.8 | DT |
| 1.55 | 10890 | 10053 | 11107 | 7.7 | 2.0 | PLSR |
| 1.70 | 10890 | 10926 | 11099 | 0.3 | 1.9 | DT |
| 1.95 | 10890 | 10607 | 10499 | 2.6 | 3.6 | DT |

**Supplementary references**

[1] P. Hess, A.H. Kung, C.B. Moore, Vibration→vibration energy transfer in methane, J. Chem. Phys. 72 (1980) 5525–5531. https://doi.org/10.1063/1.438970.

[2] L. Doyennette, F. Menard-Bourcin, J. Menard, C. Boursier, C. Camy-Peyret, Vibrational Energy Transfer in Methane Excited to 2ν3 in CH_4_ −N_2_ /O_2_ Mixtures from Laser-Induced Fluorescence Measurements, J. Phys. Chem. A. 102 (1998) 3849–3855. https://doi.org/10.1021/jp9806462.

[3] C. Boursier, J. Ménard, F. Ménard-Bourcin, Vibrational relaxation of methane by oxygen collisions: Measurements of the near-resonant energy transfer between CH_4_ and O_2_ at low temperature, J. Phys. Chem. A. 111 (2007) 7022–7030. https://doi.org/10.1021/jp072377y.

[4] C. Boursier, J. Ménard, L. Doyennette, F. Menard-Bourcin, Rovibrational Relaxation of Methane in CH_4_ −N_2_ Mixtures: Time-Resolved IR−IR Double-Resonance Measurements at 193 K and Kinetic Modeling, J. Phys. Chem. A. 107 (2003) 5280–5290. https://doi.org/10.1021/jp034265m.

[5] H.E. Bass, R.G. Keeton, D. Williams, Vibrational and rotational relaxation in mixtures of water vapor and oxygen, J. Acoust. Soc. Am. 60 (1976) 74–77. https://doi.org/10.1121/1.381050.

[6] J.N. Bass, Translation to vibration energy transfer in O + NH_3_ and O + CO_2_ collisions, J. Chem. Phys. 60 (1974) 2922–2928. https://doi.org/10.1063/1.1681461.

[7] D.L. Huestis, Vibrational Energy Transfer and Relaxation in O_2_ and H_2_O, J. Phys. Chem. A. 110 (2006) 6638–6642. https://doi.org/10.1021/jp054889n.

[8] D.R. White, Vibrational Relaxation of Oxygen by Methane, Acetylene, and Ethylene, J. Chem. Phys. 42 (1965) 2028–2032. https://doi.org/10.1063/1.1696241.

[9] H.E. Bass, Absorption of sound by air: High temperature predictions, J. Acoust. Soc. Am. 69 (1981) 124–138. https://doi.org/10.1121/1.385356.
